# A Low Cost Automatic Detection and Ranging System for Space Surveillance in the Medium Earth Orbit Region and Beyond

**DOI:** 10.3390/s140202703

**Published:** 2014-02-11

**Authors:** Radu Danescu, Anca Ciurte, Vlad Turcu

**Affiliations:** 1 Computer Science Department, Technical University of Cluj-Napoca, Memorandumului 28, Cluj-Napoca 400114, Romania; E-Mail: anca.ciurte@cs.utcluj.ro; 2 Romanian Academy, Astronomical Observatory Cluj-Napoca, Cireşilor 19, Cluj-Napoca 400487, Romania; E-Mail: vladturcu@yahoo.com

**Keywords:** space surveillance, stereo vision, automatic calibration, object detection, ranging

## Abstract

The space around the Earth is filled with man-made objects, which orbit the planet at altitudes ranging from hundreds to tens of thousands of kilometers. Keeping an eye on all objects in Earth's orbit, useful and not useful, operational or not, is known as Space Surveillance. Due to cost considerations, the space surveillance solutions beyond the Low Earth Orbit region are mainly based on optical instruments. This paper presents a solution for real-time automatic detection and ranging of space objects of altitudes ranging from below the Medium Earth Orbit up to 40,000 km, based on two low cost observation systems built using commercial cameras and marginally professional telescopes, placed 37 km apart, operating as a large baseline stereovision system. The telescopes are pointed towards any visible region of the sky, and the system is able to automatically calibrate the orientation parameters using automatic matching of reference stars from an online catalog, with a very high tolerance for the initial guess of the sky region and camera orientation. The difference between the left and right image of a synchronized stereo pair is used for automatic detection of the satellite pixels, using an original difference computation algorithm that is capable of high sensitivity and a low false positive rate. The use of stereovision provides a strong means of removing false positives, and avoids the need for prior knowledge of the orbits observed, the system being able to detect at the same time all types of objects that fall within the measurement range and are visible on the image.

## 1. Introduction

### 1.1. Motivation

The space around the Earth is filled with man-made objects, which orbit the planet at altitudes ranging from hundreds to tens of thousands of kilometers. Many of these objects—communication, navigation, military, or scientific satellites—are useful. Other objects are not so useful, and their increased number has become a threat to the safety of space operations, space travel, or even for the planet's surface, as many of these objects will, one day, fall back to Earth. These objects are also collectively known as Space Debris (SD). Keeping an eye on all the objects in Earth's orbit, useful and not useful, operational or not, is known as Space Surveillance, an activity that includes detection, tracking and propagation of orbital parameters of the space objects, followed by cataloguing and analysis. Detailed surveys on the problem of space debris and space surveillance can be found in [1,2].

### 1.2. Orbit Classification and Cataloguing

According to the Inter-Agency Space Debris Coordination Committee (IADC) [3], the space objects are categorized according to the altitude of their orbits into the following three categories: Low Earth Orbit (LEO) for an altitude below 1,500 km, Medium Earth Orbit (MEO) at an altitude of around 20,000 km, and Geostationary Earth Orbit (GEO), sometimes also called the Clarke orbit, at an altitude of 36,000 km. The Geostationary Earth Orbit and the orbits of higher altitude, such as those of the Molniya and Tundra satellites, are also known as High Earth Orbits (HEO). Another way of classifying orbits as follows: Low Earth Orbit (LEO) starts just above the top of the atmosphere, while high Earth orbit begins about one tenth of the way to the Moon [4]. In [5] we find the satellites classified as LEO (altitude less than 2,000 km), MEO (altitude between 2,000 km and 34,000 km), GEO (altitude between 34,000 km and 38,000 km), and the Remaining Earth Orbit (REO), having an altitude higher than 38,000 km.

The MEO orbit is used mainly by the navigation satellites (GPS, Glonass, Galileo), and by the communication satellites covering the North and South poles. There is no consensus about a MEO protected region [6]. The GEO protected region defined by the IADC Coordinating Committee, includes the spherical shell centered on geostationary altitude with an extent 200 km above and below this altitude and with inclination limits of +15 to −15°. While operations are usually conducted within about 75 km of geostationary altitude, the GEO protected region is extended in altitude to create a maneuver corridor for relocating spacecraft. Around 390 operational satellites orbit this protected zone, most of them providing telecommunication, broadcasting and meteorological services [7].

Several catalogs have been created and continuously updated through time in order to help and improve the process of monitoring space objects. The entity which maintains the most comprehensive catalog of satellite orbital data is the U.S. Space Surveillance Network (SSN) [8]. Other significant organizations that catalog debris and satellites are NASA, the Russian Space Surveillance System (RSSS) and the air forces from a few countries (most notably France, Germany, the UK and Canada). A future significant contribution is expected in this sense from the ESA surveillance of the space system which is currently in an early development phase [9].

### 1.3. Known Observation Systems and Processing Methodologies

The systems that are used nowadays for sky surveillance involve varied systems such as radars, optical telescopes and laser ranging systems [1]. Radar systems are successfully used for LEO objects, providing range measurements with high accuracy and, unlike optical systems, they are not sensitive to the meteorological conditions. Still, a great limitation is that they are less accurate for higher orbits. Radar suffers from 1/R^4^ signal power losses between signal transmission, reflection and reception, making detection of distant deep space spacecraft difficult. Another problem is that a radar dish able to detect such long range objects has a very small field of view (<0.20°) and the probability to detect an unknown fast moving object is insignificant.

Optical telescope systems have been employed successfully for many years to detect deep-space satellites, as they detect reflected sunlight, and therefore no active illumination is necessary for the telescope to see its target. Adding to their high performance, the optical systems are cheap and easy to set up. Moreover, the acquired data can be extensively analyzed with image processing and pattern recognition algorithms, in order to detect and track the objects of interest in real time. The main limitation is that optical systems can only operate at night, and need clear skies to acquire accurate observations. Also, the closer to Earth the orbits are (as in the case of LEO and MEO objects), the more difficult they are to detect, since they appear as fast moving objects and their background changes considerably between frames. The higher orbits (GEO, Molniya) have a significantly lower speed, being visible for a longer period of time in the same restricted area of the sky. The best known ground-based optical Space Surveillance systems are the following:

Ground Based-Electro-Optical Deep Space Surveillance-GEODSS [10] (for GEO and HEO objects) is a system of telescopes that is able to track objects as small as a basketball, orbiting the Earth at distances beyond the geosynchronous orbit. This system produces 70% of all geosynchronous tracks and 50% of all deep space tracks, tracking over 200 objects not tracked by any other sensor.

Space Surveillance Telescope [11,12], (GEO, HEO, other deep space objects) is a ground-based system using the latest in optical technology to increase the Space Situation Awareness (SSA) capability. The SST program is a DARPA technology demonstration based on a 3.5 m f/1.0 telescope, which was started in 2002, and successfully demonstrated in 2012.

The ESA Space Debris Telescope [13], designed mostly for GEO objects, operates a Ritchey-Chrétien telescope of 1 m aperture and 0.7° field of view, located in Tenerife, to detect and follow objects of at least 15 cm in diameter at GEO altitudes (assuming an object albedo of 0.1). The ESA Space Debris Telescope (ESA SDT) covers a sector of 120° of the GEO ring. Further plans by ESA include a space-based orbital debris surveillance system, able to detect much smaller objects, whose specifications are presented in [14].

ROSACE [15] is a GEO and near GEO object tracking system based on a Newton type telescope of 50 cm aperture, able to observe objects up to 19 units of visual magnitude.

The Passive Imaging Metric Sensor (PIMS) Telescopes [16] are optical systems for the surveillance of the GEO orbits and the deep space region, operated by the United Kingdom Ministry of Defense. PIMS telescopes are located in UK, Gibraltar, and Cyprus. The three sensors cover 165° of the GEO ring. The PIMS system can detect GEO objects down to 1 m diameter, with position accuracy better than 10 μrad.

The Zimmerwald Telescope [16] is a system for tracking objects in the GEO ring, operated by the Astronomical University of Berne (AIUB), based on a Cassegrain telescope with an aperture of 1 m. The sensor can cover a sector of 100° of the GEO ring. Images are taken with a CCD chip of 2,048 × 2,048 pixels, with sensitivity up to 20 mag. The Zimmerwald telescope was used as a test site for validating procedures and processing algorithms of the ESA Space Debris Telescope.

While most existing systems are focused on increasing the sensitivity by employing powerful telescopes, with narrow field of views, Space Insight's Starbrook system [17] covers a very large field of view (100 × 60°) by robotic scanning, in the pursuit of MEO objects. This system employs a fast optical system with only 10 cm aperture and a 10 megapixel CCD, and relies on automatic processing for identifying potential targets. Starbrook is currently hosted at a UK facility in Cyprus and services several UK governmental programs.

The large sky area that needs to be covered in the Space Surveillance process and the large number of resident space objects that need to be detected, tracked and identified requires automatic processing of the acquired images. A comparative study of four different methods for detection of GEO objects from CCD images is presented by Yanagisawa *et al.* in [18].

The first method is a PC-based stacking method that needs around forty CCD images to detect an object. Sub-images from consecutive frames that contain the presumed space object in exactly the same sub-image coordinates are used to create a median image. Doing so, the stars from the background are eliminated in the median image, due to the object velocity, while the space object will be emphasized. This method was able to detect several new asteroids, proving thus its effectiveness. Still, the main weakness of this method is the computational time, since it is required to process a high number of frames, and for each presumed object, there are several likely paths that must be taken into account and checked. This method is effective for known (catalogued) objects, when object's path can be predicted.

The second method is also based on stacking, sped up by using an FPGA implementation and by reducing the image complexity through a binarization pre-processing step. The computational time is therefore reduced to about one thousandth of the time required by the PC based stacking method.

The third method is a line identifying technique, which identifies the moving objects under the assumption that the pixels from consecutive frames, belonging to the same object, should fit a straight line. This method also requires multiple CCD frames for object detection.

The multi-pass multi-period (MPMP) is the fourth method, which uses the average instead of median, reducing thus the analysis time. The authors conclude that each method has its strength and weaknesses, and propose a hybrid approach for an efficient surveillance network, relying on FPGA stacking or MPMP for detection of low speed objects, on line identification for detecting fast moving objects, and on PC stacking for following objects of known trajectory, where the search area is very small.

Levesque *et al.* present in [19,20], an approach for satellite detection which relies on the property that a moving satellite will be recorded as a linear streak in a long exposure image. The method assumes that the orbital parameters of the satellite are approximately known. A matched filter is used for streak detection, requiring a detailed image analysis and several image preprocessing steps, including the correction of sensor artifacts such as dead pixels or image noise and the removal of the star bleeding or other signal degradation. Then, all the non-streak objects in the image (e.g., stars) are removed based on their morphological characteristics, thus reducing the number of possible candidates. The satellite streak detection is finally performed based on matched filters. Since the orientation and an estimative position of the satellite are known, the filter will emphasize the streak intensity and establish the real position of the satellite.

An improvement of this method is further proposed by Lévesque and Lelièvre in [21]. This new approach consists in extracting every possible object candidate and then eliminating the false positives through a set of rules based on object features. The rules were made based on a quantification of the object features such as the signal-to-noise ratio and the moments of inertia. Simulated images, generated with controlled parameters were used to establish the best thresholds for satellite streaks detection. A highly precise detection was also confirmed on real images, even in the case of very faint satellite streaks.

Stoeveken and Schildknecht discuss in [22] several methods of space debris identification in CCD images, also taking into consideration the differences between observation strategies (star tracking, target tracking or fixed orientation). The authors highlight the importance of star removal in the images based on coordinates extracted from a star catalogue, the possibility of using the median image for background subtraction, the identification of the relevant object based on specific properties (e.g., linear streak), the need for reduction of false positives caused by cosmic rays or sensor pixel errors, and the speedup in processing possible by predicting the trajectories of the objects of interest in consecutive frames.

After the orbiting object is detected in the acquired images, its orbital parameters can be computed. The classical approach for orbit computation is the method of Gauss, which requires three observations of the same object, at different moments in time. Milani *et al.* propose in [23] a new algorithm for orbit determination, based on the first integrals of the Kepler problem, which requires only two detections at different passes of the target object. The authors emphasize the need for accurate correlation between different observations of the same object, and propose sophisticated solutions for solving this problem in the case of high observation datasets. A stochastic method for orbital parameter estimation, suitable for situations where astrometrical (detection) data for a target is scarce, is presented in [24].

### 1.4. Summary of the Proposed Sensorial System and Detection Method

This paper presents a solution for real-time automatic detection and ranging of space objects of altitudes ranging from below the MEO orbit up to the apogee of the Molniya orbit, further than 40,000 km, a range which includes all GNSS satellites, the GEO satellites, and the highly eccentric orbits. The solution employs two low cost observation systems built using commercial DSLR cameras and marginally professional telescopes, placed 37 km apart, operating as a large baseline stereovision system. The telescopes are pointed towards the same specific region of the sky using the equatorial mounts. The rotation parameters are calibrated automatically with respect to an Earth-bound reference system using reference stars extracted from an online catalog, with a very high tolerance for the initial guess of the sky region and camera orientation. The system operates in star tracking mode, and its rotation parameters are continuously updated using automated matching of reference stars on the image data. The difference between the left and right image of a synchronized stereo pair is used for automatic detection of the satellite pixels, using an original difference computation algorithm that is capable of high sensitivity and a low false positive rate. The candidate pixel areas detected from the left and right image are put in correspondence using the stereo epipolar geometry, and the 3D coordinates are computed using triangulation. The use of stereovision provides a strong means of removing accidental false positives, and is able to provide instantaneous 3D data, which leads to faster determination of the orbits. Also, no assumptions are taken on the orbit of the objects observed, the system being able to detect at the same time all types of objects that fall within the measurement range and are visible on the images.

## 2. Solution Overview

### 2.1. System Initialization

The medium and high orbit object detection cycle starts with receiving the parameters of the sky region to be observed. The equatorial mounts point the two telescopes towards the specified region, and the reference stars retrieval system extracts the relevant stars that lie approximately within the observed field of view, for online rotation calibration. The telescope mounts are then programmed to compensate for Earth's motion, such that the stars in the background appear quasi stationary during the photographic exposure, and also between consecutive frames. A list of acquisition times and exposure durations is loaded into the control computer of the two observation systems, and the detection cycle can begin.

### 2.2. The Detection Cycle

The GPS receivers of the two observation systems trigger the cameras at the times specified by the acquisition schedule. Due to the fact that the GPS signals are received simultaneously in any region on Earth, the two cameras are reasonably synchronized, and thus the acquired image pair can be used for stereovision.

Reference stars, selected from the online star catalog [25], are projected into the image space, using the cameras' intrinsic parameters. For each camera, the ensemble of stars is rotated and translated so that it fits the image data. Initially, the rotation and translation search space is large enough to cover strong misalignments of angle and position between the stars in the image and the projected reference stars, but afterwards the result of the previous alignment is used as the initial search point, and the search space is drastically reduced. The matched reference stars are used for a dual purpose: to establish a warping transformation between the left and the right image, and to calibrate the rotation matrix of the cameras for the purpose of establishing the stereo geometry.

As the equatorial mount tracks the stars, the rotation matrix between the camera reference frame and the global reference frame (Earth Centered, Earth Fixed–ECEF) is continuously changing, and needs to be re-estimated. Also, the tracking process is not perfect, and the reference stars are not completely stationary between snapshots, thus excluding the possibility of automatically computing the updated rotation matrices from the Earth rotation alone. For these reasons, the rotation matrices are re-estimated at each step, using the reference stars, their matched position in the image space, the time stamp of the observation, and knowledge about the Earth's rotation.

The matched positions of the reference stars in the two images of the stereo pair are also used in the process of object detection. As the background stars do not have a detectable parallax for any baseline achievable by Earth-bound observers, we assume that these stars will have the same relative position with respect to the reference stars in both images. Based on the position of the reference stars in both images, we define warping transformations (rotation and translation) between the left and the right camera images. The right image is warped so that its star background matches the left image's, and the left image is warped to match its background to the right image's. By analyzing the difference between the original left image and the warped right image, and the difference between the original right image and the warped left one, mismatched pixels, which may be caused by orbital objects, are identified. By grouping these points through clustering using proximity criteria, candidate blobs of pixels are identified on the left and on the right image. The blobs having a significant size are selected for stereo matching.

**Figure 1.**
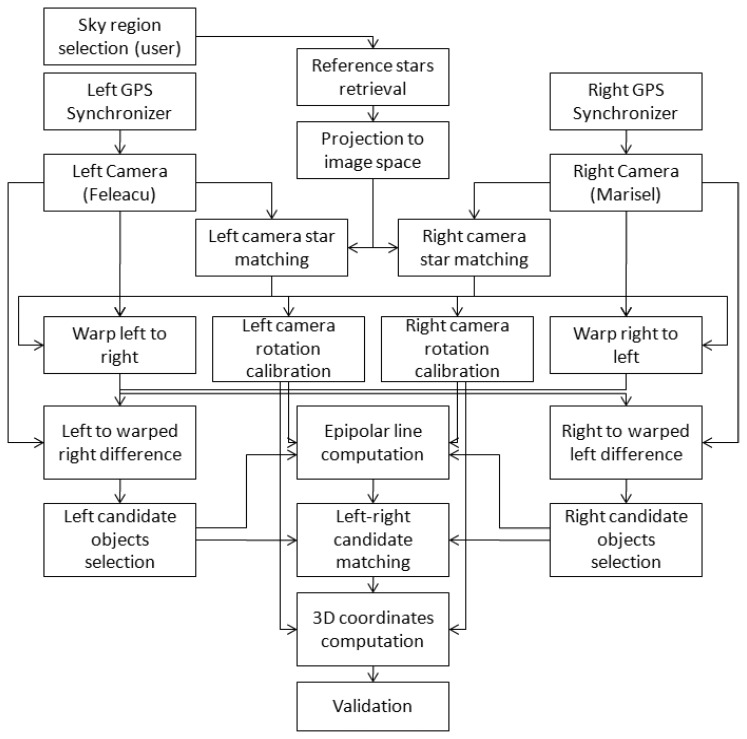
Flowchart of the calibration–detection–measurement cycle.

For each candidate blob on the left image, the epipolar line corresponding to its center of mass is computed on the right image. The intersection of this epipolar line with the right image blob's elongation axis is the correspondence point, as constrained by the stereo epipolar geometry. Based on the left-right point pair, stereo triangulation is applied and the 3D coordinates in the ECEF coordinate system are computed. If the computed distance falls within acceptable limits, the detection is validated and recorded. A graphical representation of the algorithm overview is presented in Figure 1. In the following sections, the most important steps of the algorithm will be detailed.

## 3. Sensor Setup

### 3.1. Physical Setup

The large baseline stereovision system is composed of two identical observation stations, each station having the following components:

- C6-NGT Computerized Telescope (Newtonian Reflector, D = 150 mm, F = 750 mm) [26];
- DSLR Camera, type Canon EOS 50D, with a CMOS APS-C chip (22.3 mm × 14.9 mm, 4,572 × 3,168 pixels, in color) [27];
- Equatorial tracking mount, type Celestron CG5 [26].
- Laptop computer equipped with a custom USB to TTL interface for camera triggering. The triggering is done using the cable remote interface of the camera.
- GPS Time Receiver for time synchronization and measurement of the observer's location.

The DSLR camera is connected to the telescope, which becomes the camera's objective. In order to have a decent size of the acquisition data, the 2 × 2 binning small JPEG mode of image acquisition was preferred, which produced 2,352 × 1,568 size pictures. The sensitivity of the camera was set to ISO 6400, and the exposure time was set, by trial and error, at 5 s. The equivalent angular size of one pixel, using the specified image resolution, was found to be 2.5 arc seconds, the horizontal field of view being thus 1° and 38 min, the vertical field of view 1° and 5 min, and a diagonal field of view of approximately 2 arc degrees. The entire acquisition cycle, including the time for image compression and transfer to the host computer, is 8 s. Depending on the satellite's orientation and position in the image, at this acquisition rate we are able to take between 10 and 20 images for a passing MEO satellite (approximate range of 20,000 km), and between 50 and 100 images for a passing Molniya type satellite (approximate range of 40,000 km).

### 3.2. Offline Intrinsic Calibration of the Observation Systems

For the average photographic camera, the relevant intrinsic parameters, that have to be calibrated before the camera can be used for stereovision, are the focal length in pixels (also called camera constant), the position of the principal point in the image coordinate system, and the distortion coefficients that cause the real camera to produce an image that does not comply with the theoretical perspective model that is conveniently used in computation.

Due to the fact that the telescope's field of view is extremely narrow compared to regular photographic lenses (less than 2°), we expect the radial distortions to be negligible. According to [28], for our field of view we can expect an angular precision of 0.5 arcseconds using only first order terms of the plate constants. The pixel size of our system is around 2.5 arcseconds, which suggests that the higher order distortion coefficients may be negligible. We will prove the validity of the negligible distortion assumption (for our system), by experimental data, at the end of Section 3.2.

We will also assume that the position of the principal point is not crucial in calibration, as any deviation from the true position of this point will be equivalent to a rotation of the scene with respect to the camera [29], and therefore can be compensated by the extrinsic calibration of the rotation matrix. The validity of the assumptions about the principal point will be proven in Section 7, dedicated to tests and results. Thus, the only intrinsic parameters that we need to calibrate are the focal distances: *f_L_* for the left camera, and *f_R_* for the right camera.

Given a pair of stars *i* and *j*, of known celestial coordinates and known position in the image plane, the focal distance of the camera, expressed in pixels, is:

(1) $$f(i,j)=\frac{l(i,j)}{\tan\theta (i,j)}≅\frac{l(i,j)}{\theta (i,j)}$$

The term *l*(*i*, *j*) denotes the distance in pixels between the image position of the two stars, (*x_i_*, *y_i_*) and (*x_j_*, *y_j_*):

(2) $$l(i,j)=\sqrt{{\left(x_{i}-x_{j}\right)}^{2}+{\left(y_{i}-y_{j}\right)}^{2}}$$

The term *θ*(*i*, *j*), expressed in radians, is the angular distance between the two stars. As the stars are catalogued based on their equatorial coordinates Right Ascension (RA) and declination (DEC), which are angular coordinates in a spherical coordinate system centered on Earth, the angular distance between stars *i* and *j* can be computed in spherical triangle by the cosine formula [29]:

(3) $$\theta (i,j)=\cos^{-1}(\sin({\text{DEC}}_{i})\sin({\text{DEC}}_{j})+\cos({\text{DEC}}_{i})\cos({\text{DEC}}_{j})\cos(RA_{i}-RA_{j}))$$

**Figure 2.**
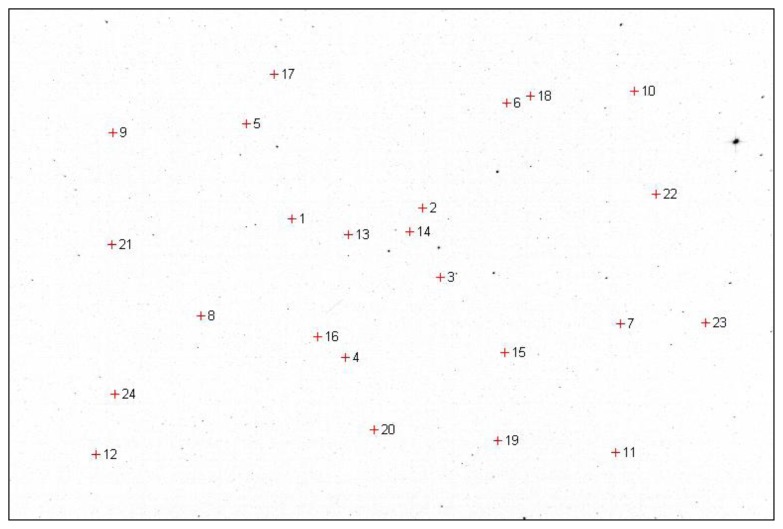
Reference stars used for internal calibration-position on the Marisel image.

The equatorial coordinates of the stars selected for calibration of the two optical systems, and an example of their corresponding position in the left and right image, are shown in Table 1. The position of these stars in the right image of the stereo pair is shown in Figure 2. The stars used for calibration are identified manually from the image, and the equatorial coordinates of these stars are extracted from a star catalog [25]. While this process may be time consuming, it must be done only once for each instrument, as the intrinsic parameters do not change during the system operation.

**Figure 3.**
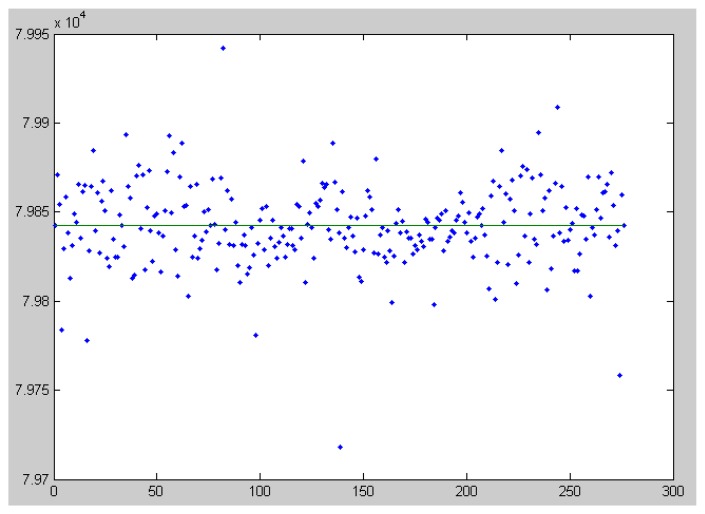
Focal distances computed from all star pairs, and the median value (Marisel).

Each pair of stars can be used for focal distance computation. By analyzing all pairs that can be formed with the available reference stars, the calibration system obtains a set of candidate focal lengths, as shown in Figure 3. For the final focal distance estimation, one can use the mean of these values, or the median. For our example, the focal distance obtained using the median is *f* = 79,839.60 pixels, and using the average the value obtained is *f* = 79,841.47. The standard deviation of the focal value candidates list is 22.71 pixels, which means that no significant outliers exist, a fact that is confirmed by the good agreement between the two estimated focal values. For comparison, a focal length value of 79,837.02 pixels was obtained using a complex astrometrical reduction process, implemented in the AIP4Win software package [30]. This indicates that the proposed calibration method is reliable and accurate, and the assumptions we relied on are valid.

As seen from Figure 3, the vast majority of the candidates are between the values of 79,800 and 79,900 pixels. If we take a point on the image that has a 1,000 pixels distance from the image center, the angle between the optical ray passing through this point and the optical axis of the camera would be around 0.01253 radians (43 min of arc), assuming a focal length of 79,800. Transforming this angle back into pixel distance, assuming the other extreme of the focal distance range, 79,900, gives us a value of 1,001 pixels. Thus, even with the most extreme range of the focal distance candidates (well outside the standard deviation interval), and a pixel position close to the borders of the image, the violation of the assumption is minimal.

In order to additionally verify the distortion magnitude, we tested linear, quadratic and cubic fits for the plate constants, using the software tool Astrometrica [31]. The linear coefficients were found to be in the order of magnitude of 10^−5^, the second degree terms were found to be in the 10^−10^ order of magnitude, and the third degree terms were lower, in the 10^−15^ domain of values. These terms lead to a radial distortion of less than 0.01 pixels at the border of the image.

The focal length computation method is run for both the left camera system (located in the village Feleacu, Cluj county, Romania) and for the right camera system (located in Marisel, Cluj county, Romania). The estimated focal distances, which are used for the rest of the stereovision process, are *f_L_* = 79,772.22 pixels, and *f_R_* = 79,839.60 pixels.

## 4. Online Calibration of the Rotation Matrices

### 4.1. The Need for a New Calibration Method

For each camera (telescope + DSLR camera rig), the extrinsic parameters are the translation vector that depicts its position in the World Reference Frame (in this case, this reference frame being the ECEF one), and the rotation matrix that quantifies the rotation between the Camera Reference Frame and the World Reference Frame. Due to the fact that the observations are carried out from fixed locations, the translation vectors are fixed, determined from accurate GPS coordinates of the two observation sites and the geodesic model of the Earth [32]. The situation of the rotation matrix is the complete opposite. Not only the rotation changes frequently, as the cameras are pointed to different regions in the sky, but as the telescopes are fixed on a tracking equatorial mount that compensates the Earth's movement so that the stars in the sky may appear to be stationary (a feature that helps in the satellite detection phase), the rotation matrices change for each frame of an observation sequence. Therefore, *the problem of extrinsic calibration becomes the problem of continuously calibrating the rotation matrices*.

The main idea, which was also described in our previous work [32], is to use the stars that are visible in the image as reference for rotation matrix calibration. As the tracking mount is not perfect, the positions of the stars will not be exactly the same in all the frames of an observation sequence, and thus the star positions have to be tracked in the image space from one frame to another. While the basic idea is still valid for the new observation setup, there are several problems that prevent us from using the same approach:

- The field of view of the telescope is much smaller than the field of view of a wide angle lens. This means that while the wide angle lens can see a large portion of the sky, allowing the possible detection of many objects, the telescope has to be frequently pointed towards new regions. This means that a set of reference stars is required for each new region that is being observed, and manually finding their initial position is time consuming and demands a highly experienced user.
- The small field of view of the telescope means that a small angular movement of the telescope mount translates in a large amount of pixel displacement of the reference stars in the image. For our setup, a pixel corresponds to 2.5 s of arc, thus even a very small, unperceivable movement can translate in tens of pixels of displacement. This situation leads to possible failures in tracking individual stars from one frame to another.
- Tracking is further complicated by the fact that a narrow field of view means that the image will be mostly populated by faint stars, of magnitude higher than 10. Tracking individual stars of such faint magnitude across frames when small jitters in the mounting rig can cause a jump of tens of pixels in the image is very difficult and unreliable.

The reasons stated above lead to the development of a new star matching method, able to quickly respond to the changing of the observed sky region, and robust in the presence of occasional jitters of the rig's position. The method is based on treating the set of reference stars as a whole object that needs to be matched to the image data. The star set object is affected by possible rotation in the image plane, quantified by a rotation angle α, and translation along the two image coordinate axes *x* and *y*, quantified by the displacements *δx* and *δy*.

### 4.2. Retrieving the Coordinates of the Reference Stars

The first piece of information required for calibration is a set of reference stars. As the observation system will be pointed towards different areas of the sky, a fresh set of reference stars must be retrieved. In order to accomplish this task, we use the U.S. Naval Observatory Interactive Catalog and Image Search [25]. The catalog allows the user to specify a sky region, in terms of the equatorial coordinates (Right Ascension and Declination) of its center, and the height and width of the region in arc minutes. Other parameters, such as the epoch of the measurement and the magnitude range of the stars to be retrieved, are provided, in order to get the most relevant stars.

We have developed a software module capable of automatically retrieving the relevant stars for any region in the sky. The approximate center of the image is specified in equatorial coordinates, along with the size of the field of view. The magnitude interval is set between 9 and 12, such that the stars do not appear too large in the image, but at the same time they should be clearly identifiable. From the list of retrieved stars, the ones that are too close together are removed, as they may cause false matching results. The remaining stars are used for calibration.

Thus, once the telescope is oriented, the equatorial coordinates of stars that can be used for calibration, *RA*_i_ and *DEC*_i_, *i* = 1…*N*, are retrieved in a matter of seconds. In what follows, we'll handle the star coordinates *RA* and *DEC* as regular angles, which means that they will be converted to radians.

### 4.3. Projecting the Reference Stars into the Image Space

After the coordinates of the stars are retrieved, a hypothetical image representation of these stars is created. This means that for each star *i* an image space coordinate pair (*x*_i_, *y*_i_) is computed.

First, the average Right Ascension and the average Declination are computed for the set of *N* calibration stars:

(4) $$\bar{RA}=\sum_{i=1}^{N}RA_{i}$$

(5) $$\bar{\text{DEC}}=\sum_{i=1}^{N}{\text{DEC}}_{i}$$

Then, relative angular displacements from the group center are computed for each star. The angular displacements corresponding to the right ascension are scaled so that the narrowing of the interval with the increase of the declination is accounted for:

(6) $$\gamma_{i}=(RA_{i}-\bar{RA})\cos(\bar{\text{DEC}})$$

(7) $$\varphi_{i}={\text{DEC}}_{i}-\bar{\text{DEC}}$$

Now, the angular displacements can be converted to pixels, by using the focal distance of the camera as a scaling factor. The resulted coordinates are centered in the image plane, by adding the half width (*w*) and the half height (*h*) of the image:

(8) $$x_{i}=\gamma_{i}f+\frac{w}{2}$$

(9) $$y_{i}=\varphi_{i}f+\frac{h}{2}$$

**Figure 4.**
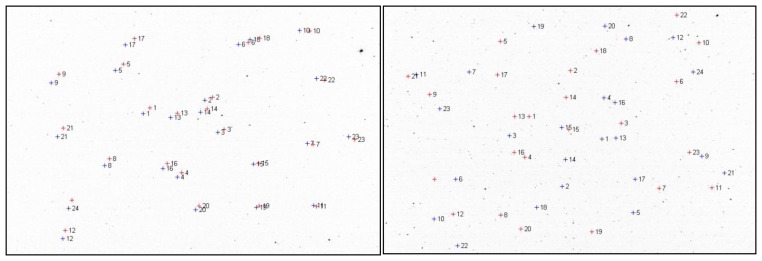
Predicted image positions of reference stars (blue) *versus* actual positions (red). Best case scenario (left, rotation of 0°), *versus* worst case scenario (right, rotation of 180°).

The predicted star coordinates in the image space can be close to the real image position of these stars, or they can be very far apart, as shown in Figure 4. The only thing one can rely on is that the relative distances between the stars in the set are correct, if the focal distance of the camera is correctly calibrated. The whole group of stars is an object that is rotated and translated with respect to its correct position in the image, and sometimes the translations and rotations can be significant (in Figure 4, right, a rotation of 180° is shown). Thus, at the beginning of an observation sequence for a specific region of the sky the star to image matching process must allow a broad range of displacement and rotation.

### 4.4. Finding the Real Position of the Reference Stars in the Image Space

The center of mass (of the star set object) has the image space coordinates *x̄* and *ȳ*, which represent the averages of the image coordinates of each star *i* in the set, *x*_i_ and *y*_i_. Applying the rotation and translation transformations to the whole star set (such that the form or the scale of the object does not change) is equivalent to altering the position of each star in the image, using the following equation:

(10) $$\left(\begin{array}{c} {x^{\prime }}_{i}(\delta x,\delta y,\alpha )\\ {y^{\prime }}_{i}(\delta x,\delta y,\alpha ) \end{array}\right)=\left(\begin{array}{cc} \cos\alpha & -\sin\alpha \\ \sin\alpha & \cos\alpha \end{array}\right)\;\left(\begin{array}{c} x_{i}-\bar{x}\\ y_{i}-\bar{y} \end{array}\right)+\left(\begin{array}{c} \bar{x}\\ \bar{y} \end{array}\right)+\left(\begin{array}{c} \delta x\\ \delta y \end{array}\right)$$

Using the transformation specified by Equation (10), the objective function that measures the quality of the match between the hypothetic star position and the acquired grayscale image *I* is defined as:

(11) $$M(\delta x,\delta y,\alpha )=\sum_{i=1}^{N}\log(I({x^{\prime }}_{i}(\delta x,\delta y,\alpha ),{y^{\prime }}_{i}(\delta x,\delta y,\alpha )))$$

In Equation (11), *N* denotes the number of reference stars. The best possible match between the start coordinates and the image is signaled by a maximum of the objective function *M*, as each star in the image has a brighter value than its surroundings. Thus, the desired parameters of the match are found as:

(12) $$\left(\delta x_{\text{match}},\delta y_{\text{match}},\alpha_{\text{match}})=\arg\max_{\delta x,\delta y,\alpha }M(\delta x,\delta y,\alpha \right)$$

The search for the best parameters for star matching is performed using a wide search space at the beginning of the observation sequence (for the *x* and *y* displacement, a range of plus or minus 300 pixels is allowed, and for the rotation angle the full 360° angular range is explored). However, searching this space is computationally expensive, and therefore in the subsequent frames of the sequence the search space is restricted around the previously found parameters. This does not exclude the possibility of failure–sometimes the movement of the rig may be too abrupt, and a re-initialization of the parameters is required. This situation is signaled by the detection routine, which will be described in the next sections. Thus, a feedback between the detection routine and the calibration routine ensures a fast star matching process, with the possibility of recovery from failure.

**Figure 5.**
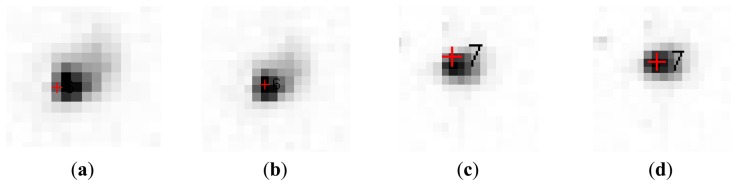
Refining the position of the stars: (**a**) and (**c**) are the positions estimated by rotating and translating the whole set of stars; (**b**) and (**d**) are the positions after local refinement.

The final step for the star to image matching process is a local refinement of the position detected by rotating and translating the whole star ensemble. In a small, 7 × 7 sized neighborhood around the estimated position, the local maximum of the intensity image is searched. Then, the positions of the pixels in the neighborhood that have a brightness equal or 5% less than the local maximum are found, and the average position obtained from these pixels is taken as the locally refined position of the star, as shown in Figure 5.

### 4.5. Finding the Rotation Matrix Using the Matched Reference Stars

For each matched star *i*, we now know its declination *DEC*_i_, its right ascension *RA*_i_, and its coordinates in the image space, *x*_i_ and *y*_i_ (for both cameras). The right ascension of the star, *RA*_i_, is converted to the Hour Angle relative to the zero meridian, *HA*_0,i_, which is the star's azimuth with respect to the zero meridian, and thus tied to the Earth Centered, Earth Fixed (ECEF) coordinate system. The relation between the Right Ascension and the zero meridian Hour Angle is the following:

(13) $$HA_{0,i}={\text{LST}}_{0}-RA_{i}$$

The term *LST*_0_ is the Local Sidereal Time of the zero meridian, obtained from the time and date of the observation.

The components *r*_n,k_, *n* = 1…3, *k* = 1…3 of the rotation matrix **R**, the declinations *DEC*_i_, the Hour Angles *HA*_0,i_, and the image positions of the stars, *x*_i_ and *y*_i_, are connected through the following equation, in which (*x*_c_, *y*_c_) is the position of the principal point and *f* is the focal distance in pixels:

(14) $$\{\begin{array}{c} \frac{x_{i}-x_{c}}{f}[r_{13}\cos{\text{DEC}}_{i}\cos HA_{0,i}+r_{23}\cos{\text{DEC}}_{i}\sin HA_{0,i}+r_{33}\sin{\text{DEC}}_{i}]+[r_{11}\cos{\text{DEC}}_{i}\cos HA_{0,i}+r_{21}\cos{\text{DEC}}_{i}\sin HA_{0,i}+r_{31}\sin{\text{DEC}}_{i}]=0\\ \frac{y_{i}-y_{c}}{f}[r_{13}\cos{\text{DEC}}_{i}\cos HA_{0,i}+r_{23}\cos{\text{DEC}}_{i}\sin HA_{0,i}+r_{33}\sin{\text{DEC}}_{i}]+[r_{12}\cos{\text{DEC}}_{i}\cos HA_{0,i}+r_{22}\cos{\text{DEC}}_{i}\sin HA_{0,i}+r_{32}\sin{\text{DEC}}_{i}]=0 \end{array}$$

For each star in the set, two equations are generated. The equation system is supra-determined, and the Gauss-Newton iterative method is used for finding the nine unknowns. More details about computing the rotation matrix when the reference stars are known can be found in [32].

## 5. Satellite Detection from Synchronized Image Pairs

The satellite streak detection in the image space follows the automatic calibration of the rotation matrices, and therefore the position of the reference stars in both images of the stereo pair is assumed to be known. Assuming that the difference in scale between the two images is negligible, as they are acquired with similar optical instruments, we can define a rotation angle and a translation vector between the left group of reference stars positions and the right group of reference stars position. Using the known image coordinates of the reference stars, the rotation angle and the translation vector (which contains the translation amounts for the *x* and the *y* coordinate) can be computed by least squares error minimization.

**Figure 6.**
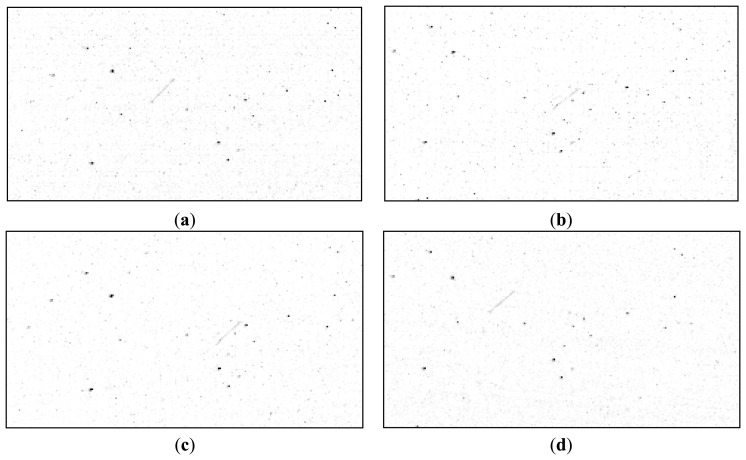
Warping: aligning the star background between the images of the stereo pair. Left (**a**) and right (**b**) original images, cropped. Warped right image to match the stars of the original left image (**c**), and warped left image to match the stars of the right image (**d**).

After the transformation parameters (rotation and translation) between the left and the right image of the stereo pair are found, they can be used to align all stars of the left image to match the ones on the right, and all the stars on the right image to match the ones on the left. This is the main idea for satellite detection: as the stars are fixed with respect to each other, transforming the whole right image using the rotation and translation parameters obtained from the reference stars analysis will get us a new image that has all the stars in the same position as the left image, but the satellite streak's position will not coincide. Figure 6 shows the warping process, for a small region of the images, containing the satellite streak. Thus, the satellite streak detection principle is the following: warp (rotate and translate) one image of the pair, keep the other image unchanged, and look for the differences.

The original image (left or right) will be considered as foreground, and the corresponding warped image (right to left, or left to right), smoothed by a convolution with a Gaussian kernel *G*, will be considered as the background. For example, for detecting the satellite pixels of the left image, the foreground and the background images are defined as:

(15) $$\begin{array}{l} I_{F}=I_{L}\\ I_{B}=I_{RL}*G \end{array}$$

**Figure 7.**
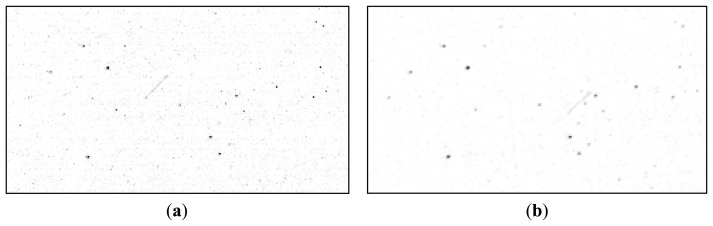
The foreground (**a**) and the background (**b**) images for satellite detection in the left image of the stereo pair.

In what follows, we'll present the detection of the satellite pixels from the left image, using the warped right image as the background, as seen in Figure 7. For detecting the satellite pixels on the right image, the same steps are applied, using as foreground the right image *I_R_* and as background the warped left image *I_LR_* convolved by the Gaussian kernel *G*.

Identifying the satellite pixels in the foreground image means identifying the relevant differences between the foreground and the background. This process is, however, more complex than simply computing the difference between the intensity values of the pixels of the two images, because the two images are acquired from different locations, with non-identical cameras, and the satellite streak signal is not very strong with respect to the stars and the noise in the image. In order to overcome these difficulties, an elaborate strategy for finding the satellite pixels was devised.

First, a threshold image *I_T_* is defined. Each pixel of the threshold image at image coordinates (*x*, *y*) is defined as a fraction of the maximum between the foreground image pixel and the background image pixel at the same coordinates:

(16) $$I_{T}(x,y)=\eta \;\max(I_{F}(x,y),I_{B}(x,y))$$

The fraction coefficient *η* is adjusted by trial and error, currently being set to *η*=0.4.

The difference between the foreground pixel intensity value and the background pixel intensity value at coordinates (*x*, *y*) is compared with the threshold image pixel value at the same coordinates. The pixel difference value above the pixel threshold indicates a possible satellite pixel at the specified coordinates. Unfortunately, this does not remove all possible false positives.

The false positives are the strong differences between foreground and background which may arise at the location of bright stars. These bright stars are not point-like in the image, and sometimes, due to chromatic aberrations or CCD saturation, they are not even circular (their shape may be elliptic, or they may have linear steaks emanating from the central point). Corrupted star shape, or uneven back lighting of the location, may lead to significant differences around the bright stars, even when their centers are aligned by warping. In order to avoid these false positives, a mask image for the regions most likely to cause false positives is created.

First, a static threshold *T* is applied to both the foreground and the background images. The value of this threshold is tuned experimentally (currently *T* = 10). The foreground mask *M*_F_ and the background mask *M*_B_ are binary (logical) 2D arrays, defined as:

(17) $$\begin{array}{c} M_{F}(x,y)=(I_{F}(x,y)>T)\\ M_{B}(x,y)=(I_{B}(x,y)>T) \end{array}$$

**Figure 8.**
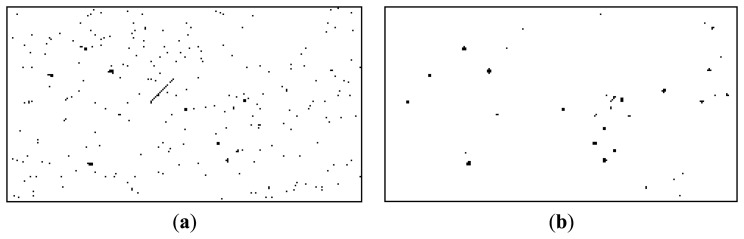
The foreground star mask (**a**) and the background star mask (**b**).

The two masks are shown in Figure 8.

**Figure 9.**
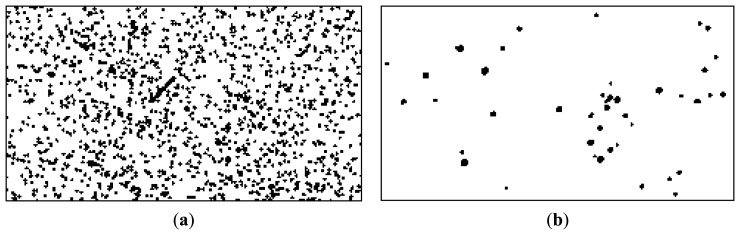
Dilated foreground star mask (**a**) and dilated background star mask (**b**).

In order to increase the safety margin, the two masks are dilated with a circular structured element *D*, with the radius of 4 pixels. This will account for possible small misalignments in the warping process, and for incomplete identification of all star pixels in the thresholding process. The result of this process consists of two dilated masks, *M*_FD_ and *M*_BD_, as shown in Figure 9:

(18) $$\begin{array}{c} M_{FD}=M_{B}⊕D\\ M_{BD}=M_{D}⊕D \end{array}$$

The two masks are then combined using a pixel wise ʌ (and) operation. The position of the bright stars is the same in the foreground and in the background, the dilation accounts for small misalignments, and thus we expect that the significantly bright stars will have non-zero (logical true) pixels in both masks. The final star mask *M* is, for each pixel position (*x*, *y*):

(19) $$M(x,y)=M_{DF}(x,y)\wedge M_{DB}(x,y)$$

**Figure 10.**
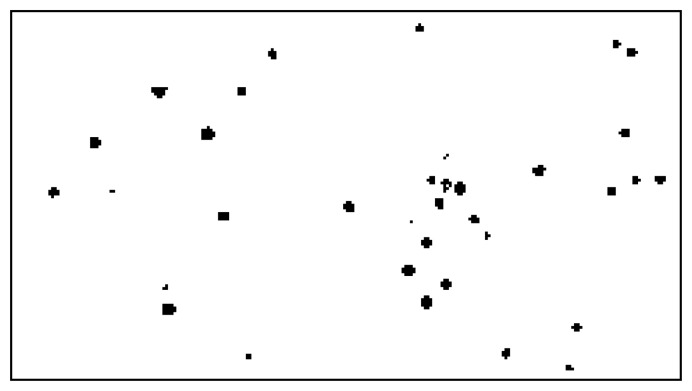
Combined star mask, showing the exclusion zones for avoiding false positives.

The final star mask is shown in Figure 10.

Now, the difference between the background and the foreground can be computed. The arithmetical difference is compared with the threshold image *I_T_*, and with the fixed, low threshold *T*, retaining the pixels that pass both thresholds, excluding those that correspond to the exclusion mask *M*. Formally, we can write:

(20) $$I_{\Delta }(x,y)=I_{F}(x,y)-I_{B}(x,y)$$

(21) IR(x,y)=(IΔ(x,y)>IT(x,y))∧(IΔ(x,y)>T)∧M¬(x,y)

**Figure 11.**
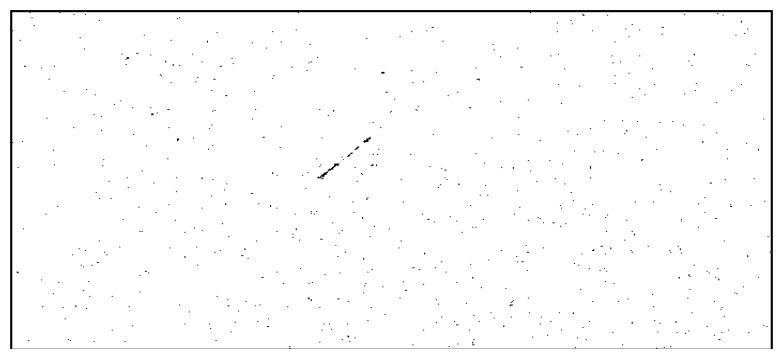
Candidate satellite pixels obtained by foreground-background difference analysis.

The result binary (logical) image *I_R_* contains all pixels for which the difference *I*_Δ_ between foreground and background passes both the global threshold *T* and the location-specific threshold *I_T_*(*x*, *y*), and do not fall inside the exclusion zone. The result image is depicted in Figure 11.

**Figure 12.**
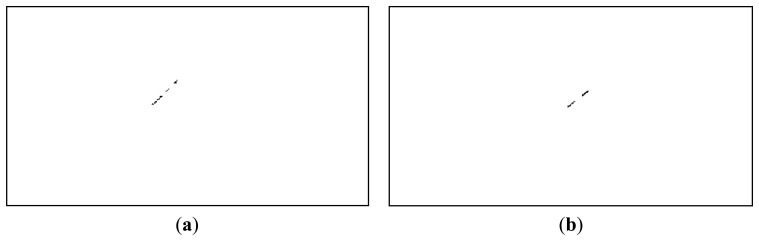
Satellite pixels, validated by area analysis. Detection results on the left image (**a**) and on the right image (**b**).

It can be seen from Figure 11 that even if the satellite's pixels are clearly identified, they are not the only pixels that have passed the established conditions. Fortunately, the other non-false pixels are usually isolated, while the pixels belonging to the satellite are grouped together into larger clusters. For this reason, a simple connected component identification process (binary image labeling) is applied, and the clusters with a pixel count of less than 10 pixels are excluded. Figure 12 shows the remaining pixels, after labeling and area based validation, for the left image as foreground (the situation for which all the processing steps have been described), and also for the right image as foreground (results obtained by executing the same algorithm steps, with the right image as the foreground and the warped left image as background).

A final step in the detection process is to cluster all streak fragments that are close together into a single object. This step is necessary because, as we can see from Figure 12, most of the streaks will not be detected as a compact object, due to the low contrast and the fact that they may be close to a significantly bright star, which will exert its exclusion zone and cut some of the pixels from the result.

This detection algorithm has a very high tolerance to noise and different background illumination between the images of the stereo pair, while retaining a high sensitivity. The number of false positives resulted from this processing stage is very low, easily removable in the next step, the stereo measurement. If a very high number of satellite candidates are found in the detection step, this is a signal that the reference stars are not matched properly in the two frames, and the star matching process is re-initialized by searching for rotations and translations in the whole parameter space, instead of reusing the position of the past frames in the sequence as the starting point, and a reduced range for rotation and translation.

## 6. Stereo Matching and 3D Coordinates Computation

**Figure 13.**
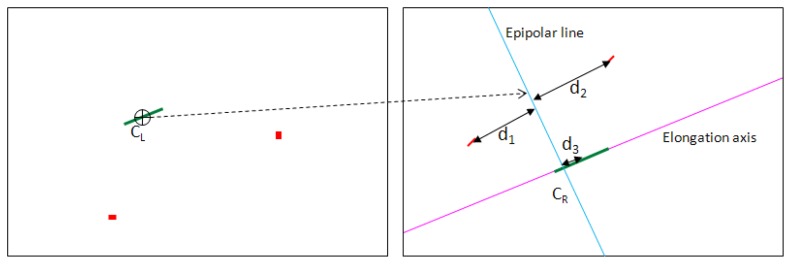
Searching for the left-right stereo correspondence. The green objects are the satellite streaks, and the red objects are the false candidates.

After the detection step, clusters of possible satellite pixels are located in both the left and the right image of the stereo pair. The clusters of the left image must be put into correspondence with the clusters of the right image, so that the stereo triangulation may be applied. The process of correspondence finding is depicted in Figure 13, and has the following steps:

**For** each candidate object on the left image:
  Compute the center of mass of the candidate object, C_L_;
  Using the fundamental matrix computed from cameras' parameters, compute the epipolar line on the right image, the geometric locus of the stereo correspondents of the left center of mass;
  **For** all candidate objects in the right image:
    Compute the distance between their centers of mass and the epipolar line (distances *d*_1_, *d*_2_, *d*_3_ in Figure 13);
    **If** the distance of a right candidate object to the epipolar line is below a threshold (*i.e.*, 50 pixels), then:
      Compute the elongation axis of the candidate object;
      Compute the intersection between the elongation axis and the epipolar line. This will be the stereo match of C_L_, denoted C_R_;
      Using C_L_ and C_R_, apply stereo triangulation and compute the 3D coordinates.
        Accept the object as valid if the distance to the observer is in the accepted range (15,000 km to 50,000 km).

Details about the epipolar line computation and stereo triangulation can be found in [32]. Using the distance to the epipolar line as a correspondence filter, and applying a range validation on the stereo 3D reconstruction results, has the effect of preventing the possible false positives of the detection stage to propagate to the final results. This is a great advantage of stereovision–imposing geometrical constraints severely limits the valid left-right pairs, and thus occasional false positives in the detection phase are not critical.

The corresponding point for the left center of mass is, as described, not the right center of mass of the object that passes the distance to the epipolar line test, but the intersection between the epipolar line and the elongation axis. This point was preferred because the center of mass is not sufficiently stable for matching–due to weak contrast or due to bright stars in the vicinity of the object, some object pixels may be lost, and the center of mass will be displaced. The elongation axis, on the other hand, does not change much, as long as an adequate number of pixels for the object are found.

## 7. Tests and Results

In order to test the proposed setup and detection method, almost three hours of observations were performed on 23 October 2012, using a synchronized setup of two sensors composed of telescope, camera, control computer and GPS antenna for time perception. One system was placed in the village of Feleacu (*Lat: N 46°42′36.50″*, *Long: 23°35′36.74″*, *Elevation: 743 m*), near Cluj-Napoca, and the other in the village of Marisel (*Lat: N 46*°*40′34.362″*, *Long: E 23*°*7′8.904″*, *Elevation: 1,130 m*), also in Cluj county. The observations covered four sky zones, specified by a central reference star, and was aimed to detect six satellites, four MEOs and two Molniyas, whose apparitions and ranges were predicted using the astronomical software The Sky [33].

**Figure 14.**
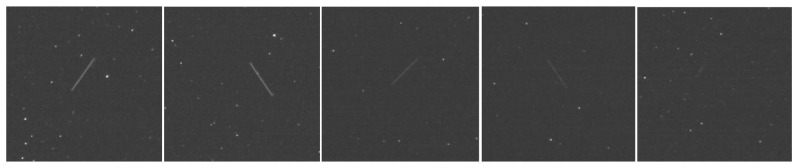
Aspect of satellites in the image space. From left to right: 733, 738, PRN10, PRN8, Molniya 3-41. Average satellites' brightness: 55, 65, 30, 22, 15 DNU. Average background brightness: 5 DNU.

The aspect of the observed satellites in the image was shown to be extremely variable. While the length of the streak was consistent with the satellite's nature, the brightness of the satellite's pixels in the image seems to be unique to each of the observed case. Some examples are shown in Figure 14: the average brightness of the satellites ranges from 15 to 65 DNU (the brightness of a pixel can have values from 0 to 255 DNU—Digital Number Unit), which makes the satellite brighter than the background, which has an average brightness of 5 DNU, but significantly less bright than the stars in the image, which can go up to the saturation value of 255 DNU. For the higher range satellites such as the Molniyas, the lower brightness is also combined with a shorter streak.

**Figure 15.**
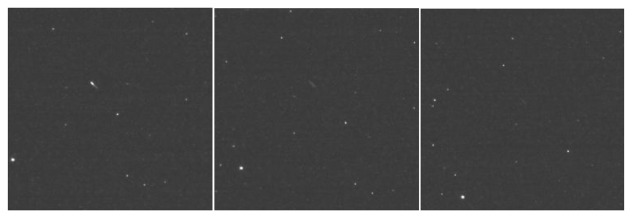
Variable perceived brightness of Molniya 1-91 due to rapid spinning. From saturation (left) to a brightness of 36 DNU (middle) and then to 12 DNU (right).

A variable brightness level, and also a variable length of the satellite streak, can be also observed for the same satellite, in different frames. The reason for this behavior is a fast spin of the satellite, which changes the amount of light the object reflects, as the reflectivity of the satellite's surface is not homogeneous. This effect is shown in Figure 15, for a Molniya type satellite, which can go from a very strong brightness (value 255 DNU-saturation) to invisibility in 3–4 frames. This behavior strongly affects the detection rate.

A summary of the detection and coordinate computation results is presented in Table 2. The predicted range, with respect to the Feleacu observation station, was generated using The Sky. The measured range is the distance between the 3D coordinate vector of the satellite, in the ECEF coordinate system, computed by stereovision, and the 3D coordinate vector of the Feleacu observation station, computed from the GPS coordinates using the geodesic model. The observation time is the time interval between the first presence of the satellite in both images of the stereo pair and the last presence. The average time elapsed between frames is 8 s, which includes the exposure time of 5 s. The detection rate is computed as the ratio between the number of frames the satellite is correctly detected and ranged and the total number of frames the satellite was observable with both telescopes.

**Figure 16.**
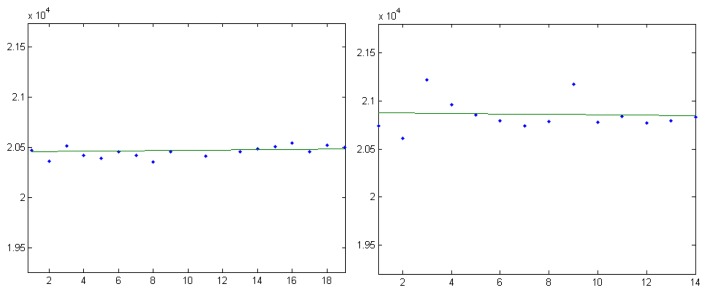
Measurement results for the observed GPS satellites, in the SAO54449 sky area. PRN 10, left, and PRN 8, right.

**Figure 17.**
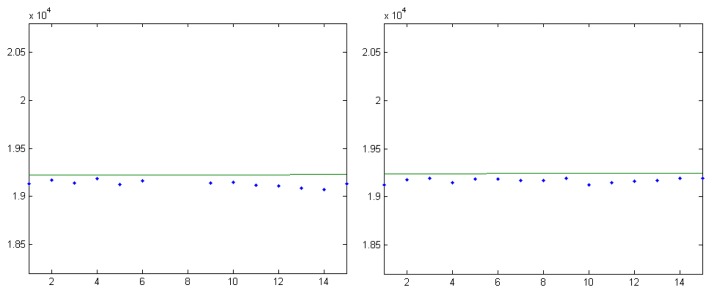
Measurement results for the observed GLONASS satellites, in the SAO37985 sky area. GLONASS 733, left, and GLONASS 738, right.

**Figure 18.**
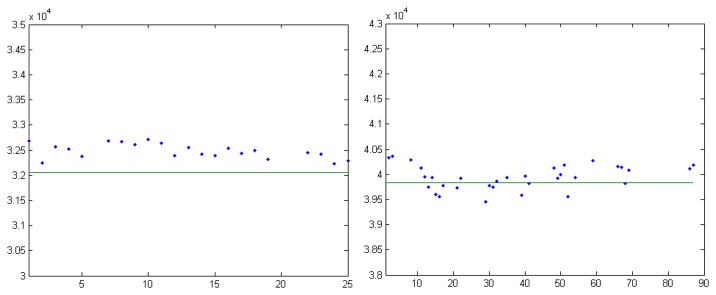
Measurement results for the observed Molniya satellites. Left: Molniya 3-41, in the SAO36361 sky region, and right, Molniya 1-91, in the SAO25214 sky region.

Frame by frame comparisons between the predicted range and the measured range are provided by Figures 16, 17 and 18. The predicted range is depicted by the continuous green line, while the measured values are presented as scattered blue dots.

The test results show variable measurement accuracy. As expected from a stereovision sensor, the accuracy is significantly better for the MEO satellites than for the Molniyas, as the accuracy drops with the distance. The measurement error corresponds to a pixel uncertainty of matching of 1–3 pixels, which, taking into account the detection method, is expected. There are, however, several cases where the measurement errors seem to be systematic, pointing to a permanent offset between the predicted position and the measurement, which may be caused by outdated predictions.

The detection rate is high for almost all satellites, and adequate for the one case that continuously goes from strongly visible to invisible. There were no false positives, and no completely false distance measurements.

We have also performed several experiments to verify the assumption that the position of the principal point is not relevant to the measurement process. The position of this point is important in itself, as it defines the interior geometry of the camera, but it does not affect the triangulation process, due to the fact that any deviation from the true value of this point will be compensated by the rotation matrices. In order to support this claim, we have significantly altered the position of the principal point, and ran the detection and measurement algorithm for the same frame, which includes a GPS satellite. We have observed the following:

- When the principal point is assumed to be in the center of the image, the satellite is detected at the ECEF coordinates (in km) X = 21,131.22, Y = 4,908.85, Z = 15,042.85, having the distance to the observer 20,278.07 km.
- When the principal point is displaced from the center by 50 pixels on both axes, the measured ECEF coordinates are X = 21,131.53, Y = 4,908.91, Z = 15,043.04, and the range is 20,278.44 km.
- When the principal point is displaced from the center by 200 pixels on the horizontal axis, and by 100 pixels on the vertical, the measured ECEF coordinates are X = 21,132.30, Y = 4,909.05, Z = 15,043.52, the range being 20,279.36 km.
- When the principal point is displaced to the top left corner of the image, more than 1,000 pixels away from the original position, the measured ECEF coordinates are X = 21,143.52, Y = 4,911.16, Z = 15,050.34, the range being 20,292.65 km.

All these displacements are extreme, well beyond the normal displacements of a principal point with respect to the image center. However, the results on the 3D reconstruction process are minor, well below the uncertainty expected from triangulation, assuming image space positioning errors of sub-pixel accuracy. Thus, our assumption about the principal point is valid.

The processing time of the current implementation (Matlab, no optimizations) is about 10 seconds per frame, on a standard PC (Intel Core i5, 4 GB RAM), which is close to real time, as the interval between frames is about 8 s. The processing cycle includes automatic calibration of the rotation matrices, satellite pixel detection in the left and right image, and stereo reconstruction–all the steps required for observation.

## 8. The Case for Stereovision-Based Detection

This paper presents a method for analyzing the difference between two images of a stereo pair, aligned in such a way that the starry background is the same, but the satellite streak, affected by the parallax, is not. Previous research, which includes our own results described in [30], relies on satellite's motion for detection. If single frame detection is required, the methods presented in the existing literature [19,20] rely on the satellite streak's shape in the image, characteristically linear.

**Figure 19.**
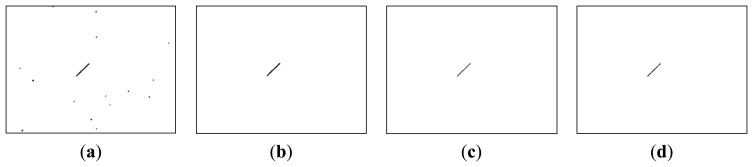
Detection of a MEO satellite, multiple methods. From left to right: (**a**) Motion detection using background subtraction; (**b**) Motion detection using background subtraction, followed by object classification; (**c**) Motion detection using the method described in this paper; (**d**) Object detection from stereo pairs, using the method proposed in this paper.

Our previous work, described in [32], relies on motion detection through background subtraction. The background is continuously updated using a running average, and the significant changes between the current frame and the background are analyzed. Due to the small angular field of view of the telescope, small imperfections of the sky tracking mechanism cause a significant number of difference areas, as shown in Figure 19a. However, if we assume that the satellite's presence is signaled by a linear streak, we can use several rules for classifying the difference regions. As shown in Figure 19b, the false positives are removed, and the satellite is correctly detected.

Unfortunately, the classification rule will only detect the MEO satellites, as shown in Table 3, column 4. Our aim is to develop a general purpose detector, for a distance range as wide as possible, and for this reason not all satellites in the detection range will present the linear streak. In fact, the most distant satellites will be undistinguishable from the false positives due to the telescope's motion, and for this reason they will not be detected using this method. Thus, if we want to detect longer range satellites, a more elaborate difference analysis method has to be employed. We have already described such a method, in Chapter 5, for the use of stereo difference analysis, but we can also use it for analyzing the difference between the current image of the sequence and the background. The method produces a low amount of false positives, as shown in Figure 19c, but is also capable of detecting both the MEO and the HEO satellites, as shown in Table 3, column 4. In fact, it seems that the proposed method for difference analysis produces similar results when applied to sequence analysis and when applied to stereo image pairs.

The experimental data shows that when our proposed method for difference analysis is applied to stereo image pairs, the results are comparable to the results obtained from motion analysis, sometimes better, *and definitely not worse*. But the stereo difference analysis has one major advantage over sequence analysis: it can produce results using images acquired at a single time instant. In a single exposure time, the system is able to detect the satellite if it is there, and to provide a decent estimate of its range, without a priori knowledge. The system can be used to scan the sky without spending much time pointed in one place, and if something is detected, more elaborate methods can be used to track it and refine its orbital parameters.

## 9. Conclusions and Future Work

This paper presented a method for Earth orbit surveillance, which relies on low cost equipment, is able to detect and range objects having an altitude from 18,000 to 40,000 km, and can do it almost in real time, with a high detection rate. The main advantages of this method are the following:

- It requires no elaborate, lengthy surveillance of the same sky region. The detection using stereovision is instantaneous–if an orbiting object is in the left and right frame, it will be detected instantly.
- It requires no assumption about the orbit to be surveyed: the same setup can detect a 19,000 km altitude satellite and a 40,000 km satellite, or both, if they happen to be in the same image.
- Automatic, instantaneous ranging: the system is able to produce a 3D coordinate vector of the object detected. While the accuracy of the measurement is not comparable to what one can obtain using multiple observations and employing elaborate orbit calculation tools, having an instant 3D position approximation may be highly valuable in the process of large sky area surveillance, and can provide a quick working estimate that can be further refined.
- Works with low cost equipment, in less than ideal working conditions. The cheap and lightweight equipment can be easily set up to a new site, and the algorithms have proven resilient to image noise and background pollution. In fact, one of the observation sites is quite close to the busy city of Cluj-Napoca, and this has not impaired the detection performance.

The method described in this paper can be easily deployed on multiple observation sites, for different types of instruments, with different focal lengths and apertures. This way, many regions of the sky can be surveyed at the same time, with detection results delivered for each acquired image pair.

## Figures and Tables

**Table 1.** Reference stars used for intrinsic calibration of the cameras.

**Equatorial Coordinates of the Reference Stars (Epoch 2012.809)**							**Left (Feleacu) Image Position**		**Right (Marisel) Image Position**	

**Star Id.**	**Right Ascension, RA**			**Declination (Dec)**						

**Nr.**	**H**	**Min**	**S**	**Degrees**	**Min**	**S**	**x**	**y**	**x**	**y**
1	1	7	59.992	30	27	15.523	897.5	637.7	871.8	643.3
2	1	9	18.150	30	23	40.995	1,291.5	569.9	1,270.6	611.6
3	1	9	34.288	30	32	23.706	1,364.1	775.1	1,324.3	822.8
4	1	8	43.397	30	44	32.087	1,099.1	1,046.7	1,035.4	1,069.2
5	1	7	24.985	30	15	35.513	732.1	360.9	732.4	352.5
6	1	10	0.519	30	8	29.159	1,517.9	226.3	1,527.5	289.7
7	1	11	27.523	30	35	21.669	1,926.1	867.9	1,876.3	966.5
8	1	7	11.636	30	41	28.684	644.1	959.1	590.2	940.3
9	1	6	4.526	30	18	59.142	326.7	426.1	322.4	380.1
10	1	11	17.184	30	4	40.327	1,905.9	154.5	1,921.0	253.4
11	1	11	35.434	30	52	12.624	1,947.9	1,260.5	1,862.2	1,359.6
12	1	6	18.646	31	1	21.167	364.9	1,410.7	270.4	1,365.2
13	1	8	35.059	30	28	23.732	1,071.7	670.7	1,042.5	692.1
14	1	9	12.212	30	26	55.228	1,258.9	643.7	1,231.3	682.3
15	1	10	19.527	30	41	4.653	1,581.3	986.1	1,521.8	1,052.6
16	1	8	24.909	30	42	15.088	1,008.9	990.3	950.7	1,004.7
17	1	7	37.711	30	8	36.944	801.7	201.7	816.2	200.0
18	1	10	14.566	30	7	9.281	1,589.5	198.5	1,601.6	268.5
19	1	10	22.933	30	52	44.634	1,586.7	1,257.1	1,502.5	1,323.5
20	1	9	6.691	30	53	24.458	1,207.1	1,257.3	1,123.9	1,288.9
21	1	6	12.019	30	33	35.802	352.9	766.3	317.5	721.4
22	1	11	38.345	30	17	45.807	1,998.5	462.5	1,985.4	568.9
23	1	12	19.396	30	33	35.771	2,186.7	838.9	2,138.3	960.9
24	1	6	25.726	30	53	7.029	406.1	1,221.3	329.0	1,179.8

**Table 2.** Measurement results for the observed satellites.

**Sky Region**	**Satellite Name**	**Observation TIME (UTC + 3)**	**Mean Predicted Range (km)**	**Mean Measured Range (km)**	**Mean Error (km)**	**Mean Absolute Error (km)**	**Detection Rate (%)**
SAO37985	GLONASS 733	0:33:24–0:35:16	19,223.60	19,131.73	−91.87	91.93	86.66
SAO37985	GLONASS 738	0:35:40–0:37:24	19,242.26	19,168.26	−74.00	73.99	100
SAO54449	GPS PRN 10	1:21:32–1:23:56	20,455.47	20,471.77	16.30	41.66	89.47
SAO54449	GPS PRN 8	1:47:48–1:49:40	20,860.97	20,849.13	−11.84	119.89	100
SAO36361	Molniya 3-41	2:09:16–2:16:04	32,054.90	32,590.26	−535.36	547.48	90.90
SAO25214	Molniya 1-91	2:37:40–2:50:44	39,828.90	39,651.32	−177.58	485.15	40.23

**Table 3.** Detection rates for multiple methods.

**Satellite Name**	**Detection Rate–Stereo Pair Difference Analysis Using Proposed Method (%)**	**Detection Rate–Motion Analysis Using Background Subtraction + Classification (%)**	**Detection Rate–Motion Analysis Using Proposed Method**
GLONASS 733	86.66	80.88	86.66
GLONASS 738	100	93.33	100
GPS PRN 10	89.47	84.20	89.47
GPS PRN 8	100	93.75	100
Molniya 3-41	90.90	**0**	81.33
Molniya 1-91	40.23	**0**	37.25
